# Risk of cadmium, lead and zinc exposure from consumption of vegetables produced in areas with mining and smelting past

**DOI:** 10.1038/s41598-020-60386-8

**Published:** 2020-02-25

**Authors:** Małgorzata Ćwieląg-Drabek, Agata Piekut, Klaudia Gut, Mateusz Grabowski

**Affiliations:** 1 Department of Environmental Health, Faculty of Health Sciences in Bytom, Medical University of Silesia, Katowice, Poland, 18 Piekarska Street, 41-902 Bytom, Poland; 2 Students Scientific Circle at the Department of Environmental Health, Faculty of Health Sciences in Bytom, Medical University of Silesia, Katowice, Poland, 18 Piekarska Street, 41-902 Bytom, Poland

**Keywords:** Environmental sciences, Risk factors

## Abstract

The study reveals links between disturbed geochemical environment being the result of mining and smelting activities with consumers exposure to toxic and carcinogenic metallic trace elements (MTEs). This study focused on evaluation on vegetable and soil pollution in family allotment gardens (FAGs), considering in the aspects of consumer exposure to cadmium, lead and zinc. Study material consisted of 219 soil samples from FAGs located in one of the most polluted areas in Poland, and 64 samples of edible plants. Contents of analyzed MTEs in topsoil in the studied area were spatially diversified and depended primarily on the location of industrial pollution sources. The average content of cadmium (0.52 mg kg^−1^ fresh weight) and lead (0.57 mg kg^−1^ fresh weight) in vegetables exceeded maximum permissible concentrations according to the European Quality Standards. Human health risk assessment was based on three scenarios of dietary exposure to cadmium, lead and zinc. In every scenario the highest average daily dose for all three elements was estimated for potatoes which are one of the main components of Poles’ diet. Presented study showed that consumption of vegetables cultivated in FAGs located in Silesia Province may pose a significant health risk for their consumers.

## Introduction

Lead and zinc mines are found all over the world. Some of them are still active, but the vast majority ended mining and processing activities years ago. However, the remains of their activities still pose a serious threat to the environment. Metallic trace elements (MTEs) such as Pb, Zn, Cd have been and are released into the environment (both natural and anthropogenic sources, e.g. volcanic eruptions, mining and extraction of different elements from their respective ores), posing a threat to the health of people exposed to them both through inhalation and food. Consumption of food (vegetables, fruits) grown in MTEs contaminated areas carries the risk of potential negative health effects^1–4^.

First family allotment gardens (FAGs) in Europe appeared at the end of the 18th century as charitable aid for city dwellers, in connection with deteriorating living conditions. Poor workers were given plots of land that they could spend on growing vegetables and fruits for their own needs^5,6^. Most often, the gardens were established in places of low attractive or peripheral districts, near industrial plants, railways and car transport routes^7,8^. In Europe, the development of allotment gardening was particularly evident in Denmark, France, Germany and the United Kingdom^9^. Allotment gardens (AGs) are still an important component of urban greenery, performing many significant functions for a city and its inhabitants. It is estimated that currently in Europe there are about three million individual allotment gardens^10^. Epidemiological studies indicate the health and social function of allotment gardens as the main functions^11^. Despite many benefits from the functioning of allotments, it should be remembered that due to their location in industrial areas of cities and along high traffic routes, they were and still are exposed to sources of industrial, communication and communal emissions, which can contribute to soil and plant contamination by metallic trace elements. Therefore, within such gardens, it would be desirable to abandon vegetable and fruit crops that may be harmful to consumers, and use them only for recreational purposes.

In Poland, allotment gardens have rich tradition with over a hundred years of history. The fastest growth of allotment gardening in Poland took place in the era of rapid urbanization in the twentieth century. In the year 1918 there were 19 gardens with 2 064 allotments on the area of 70.2 hectare. Over two decades later, in 1939, there were already 606 gardens with almost 50 000 allotments with total area of 3000 ha. After the Second World War number of gardens grew rapidly because of a huge need for allotments; in 1949 there were 1 500 gardens, with almost 120 000 allotments on the 6500 hectares. At that time, for people living in the cities, allotments were the only source of vegetables, fruits and sometimes even poultry^12^. Currently, on the 32 111.4 hectares, 4 695 gardens with 906 887 allotments are registered in Poland^13^. As in other European cities, basic function of allotments was to supply food (vegetables and fruits), especially in gardens destined to unemployed. Nowadays, in most of the European cities, one can observe a gradual replacement of the productive use of allotments by the recreational ones. However, this function is still dominant in Poland. It is estimated that close to a million families, or some 3 600 000 people, spend their leisure time on allotments^14^. It should be assumed that these are also consumers of vegetables produced there.

**Figure 1:**
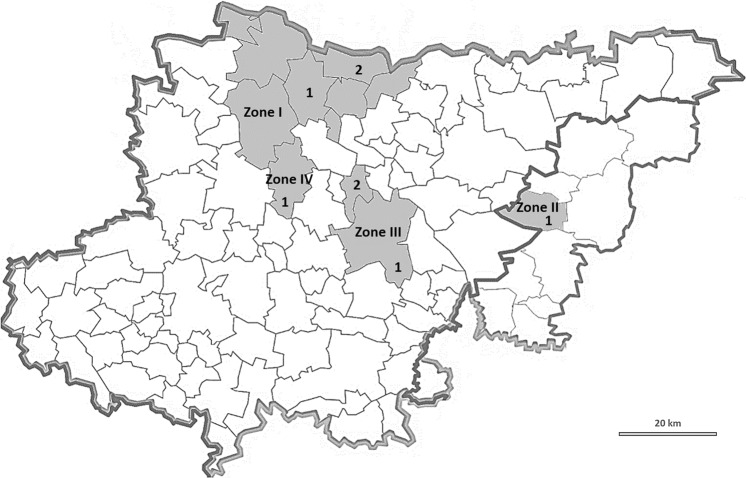
The location of sampling sites.

On a national scale, the largest number of registered family allotment gardens (FAGs) is located in the Silesia Province (662 FAGs with 10 1154 allotments)^13^. This region is at the same time one of the most polluted areas in Poland, historically related with hard coal and non-ferrous metal ores mining. The first mention of allotment gardens existing in the province come from 1893, it is an instruction for cultivating the plot of land by Upper Silesian miners and steelworkers, developed by Heinrich Kochl from Siemianowice Śląskie. The beginning of the twentieth century is the period of establishing the first allotment gardens, mainly in Chorzów, Katowice, Ruda Śląska, Toszek and Siemianowice Śląskie. The oldest FAGs existing from 1905 to the present are “A. Czarnecki” Zona III, Fig.1^15^.

The fundamental reservoir of metallic trace elements such as cadmium, lead and zinc in the environment is soil. Therefore, this environment component plays an essential role in the global metals cycles^16^. Human, as the last link in the trophic chain, is particularly exposed to the negative effects of these toxic elements. Exposure occurs through both the digestive, respiratory and dermal routes. In the general population’s environmental exposure the alimentary route plays the most important role^17^. Numerous anthropogenic activities which include coal mining, mining and smelting of metal ores, pesticide use and fertilization in agriculture and automobile exhausts are increasing the content of metallic trace elements in the environment^18,19^. Vegetables absorb MTEs mainly through the root system from a contaminated soil solution. These metals can also get into the plant through leaf blades on which they have been deposed^20^. Chronic dietary exposure to Cd and Pb is associated with various human health problems among which hematological, respiratory and cardiovascular problems are mentioned and also their impact on the development of different types of cancer^21–23^.

The Authors assume the hypothesis that consumption of vegetables grown in polluted areas of the Silesia Province may be associated with an increased health risk related to cadmium, lead and zinc exposure. The aim of the study was to assess the vegetable and soil contamination collected from family allotment gardens in selected cities of the Silesia Province, depending on their location. Vegetable contamination with metallic trace elements was considered in terms of consumer exposure to cadmium, lead and zinc. The obtained results will allow to assess the exposure level of consumers of locally grown vegetables to MTEs (Cd, Pb, Zn), which will then allow to take possible preventive steps to reduce health risk.

## Materials and Methods

### Study area

The study area comprised allotment gardens situated in the region of Silesia Province (50°20′N and 19°00′E), in southern Poland, centered on the historic region known as Upper Silesia (Fig. 1).

The Silesia Province is one of the most contaminated region of Poland, which is resulted by many years of industrial activity like mining or metallurgy of zinc and lead. Negative effects of mining activity are especially recognizable in the area of the Upper Silesian Coal Basin, where for over 150 years more than 13 billion tons of various mineral raw materials have been extracted^24,25^. Katowice, which is also the capital of the province, had at the end of the eighties in the 19th century a powerful mining and metallurgy center with coal concern “Kattowitzer Aktien-Gesellshaft”, Miners’ Guild and Upper Silesian Coal Convention^25^. An important industrial facility in Katowice was also Non-Ferrous Metalworks “Szopienice” (1834–2008), which at the beginning of the 20th century became the largest producer of non-ferrous metals in Silesia and the largest center of cadmium production in the world^26^. Soils in the neighborhood of metal mines and smelters showed metal content exceeding background up to 100-times^27^. One of the most polluted city in the entire province is the city of Chorzów (Zone III, Fig. 1), where the average life expectancy of inhabitants is the lowest in the entire province^28^. Long-term activity of such industrial facilities as Royal Iron Works (later “Kościuszko” Iron Works, 1802-still), “Lidognia” Zinc Works (1809–1899), Royal Coal Mine (1870–1970) and “Batory” Iron Works (1873-still) is associated with significant pollution of soils with metallic trace elements. In Zone IV, the main sources of pollution to the environment were mainly “Zabrze” Iron Works (1972-still), “Guido” Coal Mine (1871–1928) and “Makoszowy” Coal Mine (1906–2016)^29,30^. The area of Zone I (Fig. 1) is known for historic lead-ore mining with a significant contribution to the global production of this metal. The lead-ores mined in this city contain quite a big admixture of silver. Tarnowskie Góry Lead-Silver-Zinc Mine and its Underground Water Management System were in 2017 inscribed on the UNESCO World Heritage List^31^. There is still operating zinc smelter in Zone I (sampling point 2). In the vicinity of the Zinc and Lead Smelter, forest and other vegetation have been almost completely destroyed. During the 1970s and 1980s, the smelter was a major source of atmospheric emissions of lead and other metallic trace elements in the northern part of the Silesia Province^24^. The Mining and Metallurgical Plants “Bolesław” in Zona II (Fig. 1) were created in 1955 after the merger formerly operating smelter and mine with the same name. The investigations of the metallic trace elements contents in the soils in the vicinity of a Zn-Pb mining and smelting complex have shown high content of Zn, Pb and Cd (234–12 400; 42–3 570; 25–l33 mg/kg, respectively)^32,33^.

### Sampling

The study material consisted of 219 soil samples from family allotment gardens located in four zones of the study area and 64 samples of edible plants, including carrot, potato, beetroot, parsley and celery. Vegetable samples were collected during years 2013–2017, from crops already grown in the allotment gardens, according to their availability in each site. The samples included the most popular vegetables in Polish diet (Table 1). The subject of our interest were FAGs, due to the fact that users of these gardens and their families are at the same time also consumers of vegetables produced there.

**Table 1:** Description of analyzed vegetable samples.

Common name	Scientific name	Edible part
carrot	*Daucus carota L*.	root
potato	*Solanum tuberosum L*.	tuber
beetroot	*Beta vulgaris L*.	bulb
parsley	*Petroselinum crispum L*.	root
celery	*Apium graveolens L*.	root

The arable layer of soil was collected using the accidental sampling method. At each sampling site, soil samples were collected with the use of a hand-held twisting probe (Egner’s stick), from 10 to 15 holes in the surface of 1m^2^. Soil collected from the depth of 0–20 cm was used as material for analysis. Each time 10–15 individual samples were taken thereby providing a mean mixed sample about 500 g of the soil. The samples of vegetables and soils were collected from the same sampling points from allotment gardens.

### Samples preparation and chemical analyses

Soil samples were dried in electric oven, model WG-71 Chemland (Poland) at temperature 105 °C and sifted through a sieve with a diameter of <2 mm. Samples of 500 mg were then prepared using analytical balance, model AS60/220/C/2, Radwag (Poland). Vegetable samples were prepared for extraction the same way they are prepared for consumption: some non-edible parts of the vegetables were removed, and then they were washed in order to remove soil particles. Vegetables like carrot, potato, beetroot, parsley and celery have been peeled and shredded. Finally, 1 g of fresh mass of each vegetable was weighed using analytical balance (Radwag). Supra pure grade reagent (Merck, Germany) of nitric acid was utilized for the digestion of soil and vegetable samples in a Teflon vessel using microwave reactor, model Magnum II (Ertec, Poland) with computer control of pressure and temperature. The 0.5 g of dry soil samples were digested for 10 min in 8 ml of nitric acid, at the 42–45 bar pressure, and 1 g of fresh vegetable samples were digested for 7 min in 10 ml of nitric acid, at 42–45 bar pressure.

The content of cadmium, lead and zinc in the analyzed soil and vegetable samples were determined by the method of atomic emission spectroscopy with inductively coupled plasma (ICPOES) using the spectrometer model Integra XL (GBC, Australia). The samples with the low metal content were again determined by Atomic Absorption Spectrophotometer (AAS) Savant AA Sigma (GBC, Australia). The content of analyzed metals in the samples of soils was calculated on the dry weight basis, while in the vegetables was calculated on fresh weight.

The results of metallic trace elements content were validated using Certificate of Reference Material 1000 mg l^−1^ Lead Matrix: 2% HNO_3_ SPEX CertiPrep and Certificate of Reference Material 1000 mg l^−1^ Cadmium Matrix: 2% HNO_3_ SPEX CertiPrep standard solutions. Quality control measures: standard reference samples (Certificate of Certified Reference Materials NCS ZC73012 Cabbage from the China National Analysis Center for Iron and Steel) in vegetables and Analytical Reference Material Soil S-1 (Department of Physics and Nuclear Technology at the Academy of Mining and Metallurgy in Cracow) were used in metallic trace elements analyses. The pH of the soil samples was measured using pH-meter model CPC-401 (Elmetron, Poland).

### Health risk assessment

The daily dietary intake of cadmium, lead and zinc by residents of study area was calculated for health risk assessment using the data from market analysis prepared by the Institute of Agricultural and Food Economics – National Research Institute^34,35^, US EPA^36^ and Roba *et al*.^37^. According to the Commission Regulation No 420/2011 and No 488/2014 setting maximum levels for certain contaminants in foodstuffs, the maximum allowable concentrations of analyzed metals in the food were used for all the scenarios^38,39^. Results of contamination with cadmium, lead and zinc of six vegetable species were included in second (average content) and third scenario (highest content).

The Average Daily Dose (ADD) of ingested Cd, Pb and Zn by adult resident of analyzed area, depending on scenario, according to recommendation of the US EPA^36^, was calculated using the following equation:

$${\rm{ADD}}={\rm{C}}\times {\rm{IR}}\times {\rm{EF}}\times {\rm{ED}}/{\rm{BW}}\times {\rm{AT}}$$

ADD is the Average Daily Potential Dose of metallic trace element through ingestion (mg kg^−1^ day^−1^); C is the metal content in the food product (mg kg^−1^); IR is the ingestion rate (kg day^−1^); EF represents the exposure frequency (365 days^−1^); ED is the exposure duration (70 years); BW is the body weight (70 kg); AT represents the average exposure time (EF × ED). Hazard quotient (HQ = ADD/RfD) was used to calculate non-carcinogenic risk for humans by ingestion of food. The hazard quotient less than 1 is assumed to be safe, equal to or exceeding 1 may concern potential non-carcinogenic effects. RfD (reference dose) is the tolerable daily intake of the contaminant (mg kg^−1^ day^−1^) via the oral exposure. The RfD for Cd was based on 1 × 10^−3^ mg kg^−1^^40^. The RfD for Pb has not been currently established by US EPA^41^, therefore, RfD for Pb in this study was 3.6 × 10^−3^ mg kg^−1^, calculated from the tolerable weekly Pb intake limit (25 μg kg^−1^) recommended for adults by the FAO/WHO^42^. The reference dose for Zn was 0.3 mg kg^−1^ ^43^.

Health risk assessment was based on the three exposure scenarios. In the first scenario was assumed that the content of cadmium and lead in all vegetable species was equal to the maximum allowable concentration according to the Commission Regulation (EU) No. 488/2014 (for cadmium) and No. 420/2011 (for lead). In 1982, JECFA (the Joint FAO/WHO Expert Committee on Food Additives) proposed a provisional maximum tolerable daily intake (PMTDI) for zinc of 1 mg kg^−1^ of body weight, however, taking into account recent studies on humans, the derivation of a guideline value is not required at this time. The daily requirement of zinc for adult men is 15 mg day^−1^ which, in this study, was taken as the maximum allowable concentration^44^. The second scenario took into account the average content of metallic trace elements determined in analyzed vegetables, while in the third scenario the health risk was assessed using the maximum determined content of Cd, Pb and Zn.

## Results and Discussion

### Soil contamination

The content of metallic trace elements (Cd, Pb and Zn) in topsoil of allotment gardens in the studied area was spatially diversified and depended primarily on the location of industrial pollution sources. Similar results were obtained in the study conducted by Kabala *et al*.^45^. The average content of cadmium, lead and zinc in the vast majority of the analyzed soil samples collected from allotment gardens exceeded maximum permissible concentrations (MPCs) according to the National Quality Standards (Cd – 3 mg kg^−1^ dry weight, Pb – 250 mg kg^−1^ dry weight, Zn – 500 mg kg^−1^ dry weight)^46^. Excessive rate refers to the ratio of tested samples that exceeding the national standard limits. All collected samples in Zone III (sample point 2) exceeded the MPCs for cadmium and lead. The obtained ranges of cadmium, lead and zinc in the analyzed soil samples from all sites were <2.0–69.9, <20.0–2 823.9, 280.7–7 443.0 mg kg^−1^, respectively. The highest average soil content of Cd and Pb were found in the samples collected from Zone III, point 2 and Zone I, point 2 while the lowest in Zone IV, point 1 (Table 2).

**Table 2:** Cadmium, lead and zinc content in soil samples from family allotment gardens in the selected study areas.

Zone	Sampling points	Number of soil samples	Element [mg kg^−1^ dry weight]	Mean ± SD	Min	Max	Median	Excessive Rate (%)
I	1	50	Cd	6.1 ± 3.0	<LOQ	12.9	6.2	84.0
			Pb	423.5 ± 224.4	115.2	1009.1	409.4	68.0
			Zn	1283.3 ± 733.3	318.4	3218.8	1202.6	90.0
	2	40	Cd	13.6 ± 14.1	2.8	69.9	8.8	97.5
			Pb	649.4 ± 458.7	179.9	2777.6	508.9	97.5
			Zn	1147.7 ± 1157.1	280.7	7443.0	870.7	92.5
II	1	7	Cd	11.2 ± 4.8	5.8	18.2	10.9	100.0
			Pb	463.8 ± 352.5	152.3	1224.4	403.2	85.7
			Zn	2254.7 ± 2046.0	705.4	6736.8	1888.0	100.0
III	1	27	Cd	9.1 ± 4.4	2.5	18.3	9.2	96.3
			Pb	284.9 ± 152.4	<LOQ	659.0	258.8	51.9
			Zn	1258.8 ± 816.4	375.8	4008.0	1039.1	92.6
	2	49	Cd	14.8 ± 3.4	8.8	33.1	14.8	100.0
			Pb	1903.9 ± 314.2	1302.2	2823.9	1903.3	100.0
			Zn	468.5 ± 91.6	325.4	772.2	470.2	36.7
IV	1	48	Cd	2.2 ± 1.2	<LOQ	5.3	1.8	20.8
			Pb	100.2 ± 35.1	42.5	212.0	93.5	0.0
			Zn	—	—	—	—	—
MPC			Cd	3				
			Pb	250				
			Zn	500				

The pH value in the analyzed soil samples varied from 6.2 to 8.5 (Table 3). The bioavailability of cadmium, lead and zinc from contaminated soils to plants largely depends on soil acidity^47,48^. The transfer of metallic trace elements ions from the soil into the plants is diminished at a pH greater than 6.5. This value has not been exceeded only in a few soil samples collected from allotment gardens located in Zone III, point 1 (Fig. 1). In all sampling sites the average pH values of soil were greater or equal to 7.6 (slightly alkaline). Soils with neutral and alkaline reactions decrease the mobility of MTEs, therefore, agricultural practices aimed at reducing the transfer of metallic trace elements from soil to edible plants, as liming, are not recommended.

**Table 3:** The pH of soil samples from family allotment gardens in the selected cities of study area.

Place of sampling	Number of soil samples	Soil pH		
		Mean	Min	Max
TG	50	7.7	7.1	8.4
MS	40	7.6	6.9	8.4
KT	27	7.6	6.2	8.4
CH	49	7.7	7.1	8.4
ZA	48	7.7	6.6	8.5
BU	7	8.0	7.7	8.2

### Cadmium, lead and zinc content in vegetables

The average content of cadmium and lead in vegetables from analyzed allotment gardens exceeded maximum permissible concentrations (MPCs) according to the European Quality Standards^38,39^ (Table 4). Permissible concentration of cadmium was exceeded over 8 times in carrots, 5 times in celeries, 3 times in beetroots and about 2 times in parsleys and potatoes. The maximum permissible concentration for lead was exceeded over 11 times in parsleys, almost 8 times in carrots, 4 times in beetroots and celeries, and 2 times in potatoes. High content of these two metals were recorded in carrots. The average content of cadmium in the analyzed vegetables decreased in following order: celery>carrot>beetroot>parsley>potato, while lead content decreased in order: parsley>carrot>celery>beetroot>potato. Content of zinc in analyzed vegetables decreased in order: beetroot>celery>parsley>carrot>potato.

**Table 4:** Cadmium, lead and zinc content in vegetables cultivated in the study area.

Vegetable	N	MPC mg kg^−1^ fresh weight			Cadmium mg kg^−1^ fresh weight			Lead mg kg^−1^ fresh weight			Zinc mg kg^−1^ fresh weight		
		Cd	Pb	Zn	Mean	Min	Max	Mean	Min	Max	Mean	Min	Max
carrot	14	0.10	0.10	—	0.85	0.04	4,82	0.78	<0.007	2.12	16.22	6.02	28.34
potato	16	0.10	0.10		0.18	0.01	1.70	0.20	0.05	1.65	12.27	0.03	161.56
beetroot	8	0.10	0.10		0.30	0.07	0.59	0.37	<0.007	0.85	58.77	44.86	72.18
parsley	13	0.10	0.10		0.25	0.01	0.69	1.11	<0.007	5.54	31.98	11.86	57.71
celery	13	0.20	0.10		1.00	0.10	4.54	0.41	<0.007	1.77	54.01	26.15	89.48

### Health risk assessment

Human Health Risk Assessment presented in this study was based on three scenarios of dietary exposure to cadmium, lead and zinc (Table 5). In every scenario the highest average daily dose (ADD) for all three metals was estimated for potatoes. The calculated Hazard Quotient (HQ) for cadmium and lead except zinc, in scenario I and II, separately for each species of vegetables, was found to be below 1 which indicates safe with no risk to human health. Higher HQ value for Zn in all scenarios may not pose risk to human health because it is an essential trace element. The third scenario assumed that the content of cadmium, lead and zinc in five analyzed species of vegetables were equal to the maximum content found in the study. In this scenario HQ over 1, when potential non-cancer effects may occur, was estimated in case of cadmium dietary exposure for two vegetable species; HQ over 3.5 was obtained for potato, 1.2 for carrot. Hazard quotient close to 1 (0, 95) was calculated for potato in case of lead dietary exposure. Content of Cd and Pb in the potato samples were the lowest among all analyzed vegetable samples. However, the ingestion rate (kg day^−1^) in the analyzed population was the highest in case of this vegetable which had a direct impact on the hazard quotient value. Considering the simultaneous daily consumption of all of the listed vegetable species, HQ above 1 was recorded for Cd in I, II and III scenario, while for lead in scenario II and III.

**Table 5:** Average daily dietary exposure to cadmium, lead and zinc (mg kg^−1^ day^−1^) and hazard quotient (HQ) in three exposure scenarios.

Vegetable	Daily exposure								
	Scenario I			Scenario II			Scenario III		
*Average Daily Dose*	Cd	Pb	Zn	Cd	Pb	Zn	Cd	Pb	Zn
carrot	0.00002	0.00002	0.00364	0.00021	0.00018	0.00394	0.00117	0.00051	0.00688
potato	0.00021	0.00021	0.03107	0.00037	0.00041	0.02542	0.00352	0.00342	0.33466
beetroot	0.00001	0.00001	0.00171	0.00003	0.00004	0.00672	0.00007	0.00010	0.00825
parsley	0.00001	0.00001	0.00154	0.00003	0.00011	0.00329	0.00007	0.00057	0.00594
celery	0.00002	0.00001	0.00150	0.00010	0.00004	0.00540	0.00045	0.00018	0.00895
*Total*	*0.00158*	*0.00132*	*0.19736*	*0.00679*	*0.00747*	*0.45590*	*0.03247*	*0.03139*	*1.07696*
***Hazard Quotient***									
carrot	0.02429	0.00675	0.01214	0.20643	0.05060	0.01313	1.17057	0.14302	0.02294
potato	0.20714	0.05754	0.10357	0.37286	0.11508	0.08472	3.52143	0.94940	1.11553
beetroot	0.01143	0.00317	0.00571	0.03429	0.01175	0.02239	0.06743	0.02698	0.02750
parsley	0.01029	0.00286	0.00514	0.02571	0.03171	0.01096	0.07097	0.15829	0.01979
celery	0.02000	0.00278	0.00500	0.10000	0.01139	0.01800	0.45400	0.04917	0.02983
*Total*	*1.57886*	*0.36548*	*0.65786*	*6.78909*	*2.07590*	*1.51965*	*32.47183*	*8.72026*	*3.58988*

*The assumptions were based on the example of the study of Dziubanek *et al*.^33^.

Although, the HQ-based risk assessment method does not provide a quantitative estimation for the probability of an exposed population experiencing a reverse health effect, it factually provides an indication of health risk level due to exposure to pollutants such as metallic trace elements^49,50^.

In the non-smoking population the dominant source of human exposure to such metallic trace elements as Cd and Pb is food. In a number of studies levels of these metals have been investigated in various food products^51,52^. The major pathway of human exposure to metallic trace elements is their transfer from soil to edible plant. Plants take up metallic trace elements and accumulate them in their edible and non-edible parts. Consumed in sufficiently high amounts can cause human health problems^50^.

Cadmium is statistically associated with an increased risk of cancer. This element is nephrotoxic and may cause kidney failure. It also participates in the process of bone demineralization. According to the European Food Safety Authority (EFSA) the greatest impact on dietary exposure to cadmium had food consumed in larger quantities; this was true for grains and grain products (26.9%), vegetables and vegetable products (16.0%), starchy roots and tubers (13.2%). It was confirmed that potatoes (13.2%), bread and rolls (11.7%), fine bakery wares (5.1%), chocolate products (4.3%), leafy vegetables (3.9%) and water molluscs (3.2%) contributed the most to cadmium dietary exposure in all age groups^53^. The mean dietary exposure to cadmium for adults across Europe is close to, or slightly exceeding, the tolerable weekly intake (TWI for Cd − 2.5 μg kg^−1^ b.w.). Vegetarians, children and people living in highly contaminated areas may exceed the TWI by about 2-fold^54^.

Exposure to lead occurs mainly through the food chain, although ingestion of soil and dust can also be an important contributor. In recent years, the long-term exposure to Pb through food intake, which can cause adverse health effects (even at relatively low levels), has been intensively studied for human health risk assessment^55,56^. Exposure to Pb can impair brain and nervous system, and can also cause chronic kidney disease. Based on middle bound mean lead occurrence it was estimated, that mean lifetime dietary exposure to lead in the overall European population amounts to 0.68 μg kg^−1^ b.w. per day. Important food category contributors in dietary exposure to Pb include: bread and rolls (8.5%), tea (6.2%), tap water (6.1%), potatoes and potato products (4.9%), fermented milk products (4.2%) and beer and beer-like beverages (4.1%)^57,58^.

Many adverse health effects of metallic trace elements have been known for a long time. However, this is not a problem from the past - exposure to this contaminants continues^59^. Potential public health risks resulting from dietary exposure to metallic trace elements, such as cadmium and lead, continue to be the significant subject of research, regulation and debate. In every Polish city, part of the land is used as Family Allotment Gardens, where decorative plants are grown as well as fruits and vegetables for consumption. Because of their central location in a city or along heavy-traffic roads further functioning of allotment gardens in the present form (cultivation of vegetables) is becoming increasingly controversial^14^. It is extremely important to inform the local community about the existing threat and about possible preventive actions that will effectively manage individual health risks in an environment contaminated with metallic trace elements.

The main cause of vegetables contamination with metallic trace elements grown in allotment gardens located in Silesia Province in Poland may be the topsoil pollution by cadmium and lead. Presented study showed that consumption of vegetables cultivated in FAGs located in Silesia Province may pose a significant health risk for their consumers. Study shows that the crucial preventive measure to high metallic trace elements exposure in Family Allotment Gardens located in areas with mining and smelting past is resignation from growing plants that are an important part of the diet of garden users. The recommended preventive measure in this case is the replacement of vegetable crops with ornamental plants, or instead of vegetables that strongly accumulate metallic trace elements, the introduction of species that accumulate metals to a small extent, such as onion or garlic^60^. Vegetables with a large share in the daily diet of the inhabitants of a given region, such as potatoes or carrots, should be obtained from unpolluted places in Poland, Europe or the world. The results obtained will be presented to local government units, appropriate for the particular studied area, in order to present them as well as possible preventive recommendations to allotment gardens users.

## Supplementary information

Supplementary dataset .
