# Supplementary material for: Risk of cadmium, lead and zinc exposure from consumption of vegetables produced in areas with mining and smelting past
Source: Sci Rep. 2020 Feb 25;10:3363. doi: 10.1038/s41598-020-60386-8 (PMC7042296; doi:10.1038/s41598-020-60386-8)
Supplement: Supplementary file 1 — Supplementary dataset . [file 41598_2020_60386_MOESM1_ESM.docx]

**Risk of cadmium, lead and zinc exposure from consumption of vegetables
produced in areas with mining and smelting past**

Małgorzata Ćwieląg-Drabek^1^, Agata Piekut^2^, Klaudia Gut^2^, Mateusz Grabowski^3^

^1^ Department of Environmental Health, School of Health Sciences in Bytom, Medical University of Silesia in Katowice (Poland), 18 Piekarska Street, 41-902 Bytom, Poland

^2^ Department of Environmental Health, School of Health Sciences in Bytom, Medical University of Silesia in Katowice (Poland), 18 Piekarska Street, 41-902 Bytom, Poland

^3^ Students Scientific Circle at the Department of Environmental Health, School of Health Sciences in Bytom, Medical University of Silesia in Katowice (Poland), 18 Piekarska Street, 41-902 Bytom, Poland

**Corresponding author**: M. Ćwieląg-Drabek. Department of Environmental Health, School of Health Sciences in Bytom, Medical University of Silesia in Katowice (Poland), 18 Piekarska Street, 41-902 Bytom, Poland. E-mail: mdrabek@sum.edu.pl. Telephone: +48 323976529. ORCID: 0000-0002-429-9949.

**SUPPLEMENTARY TABLES**

**Table 6** Content of Cd, Pb and Zn in soil samples and soil pH from family allotment gardens in **zone** **No. I**, **sampling point No. 1**

| Sample number | Cd  [mg kg^-1^ dry weight] | Pb  [mg kg^-1^ dry weight] | Zn  [mg kg^-1^ dry weight] | pH |
| --- | --- | --- | --- | --- |
| 1 | 6.2 | 579.0 | 1192.9 | 8.0 |
| 2 | 5.6 | 475.4 | 1366.9 | 8.0 |
| 3 | 6.3 | 492.1 | 1604.6 | 8.0 |
| 4 | 4.5 | 244.8 | 813.5 | 7.7 |
| 5 | 2.9 | 129.4 | 501.9 | 8.2 |
| 6 | 3.5 | 410.0 | 1023.2 | 7.7 |
| 7 | 3.1 | 246.2 | 638.9 | 8.0 |
| 8 | 7.6 | 529.6 | 1410.5 | 8.4 |
| 9 | 2.5 | 198.1 | 643.4 | 7.8 |
| 10 | 0.0 | 119.3 | 397.8 | 7.9 |
| 11 | 4.2 | 329.5 | 853.2 | 7.6 |
| 12 | 6.0 | 232.5 | 1096.7 | 7.8 |
| 13 | 8.7 | 226.7 | 1386.4 | 7.5 |
| 14 | 4.3 | 292.7 | 730.5 | 7.8 |
| 15 | 10.8 | 1009.1 | 2899.0 | 7.9 |
| 16 | 10.3 | 969.9 | 1428.3 | 7.9 |
| 17 | 6.2 | 656.9 | 1582.0 | 7.5 |
| 18 | 7.6 | 834.7 | 1730.3 | 7.6 |
| 19 | 7.4 | 722.6 | 1818.4 | 7.3 |
| 20 | 8.9 | 840.5 | 2332.2 | 7.5 |
| 21 | 6.7 | 529.1 | 1471.1 | 7.3 |
| 22 | 9.3 | 612.7 | 2174.7 | 7.1 |
| 23 | 12.7 | 597.3 | 2671.7 | 7.2 |
| 24 | 12.1 | 411.3 | 2420.1 | 7.8 |
| 25 | 12.9 | 538.1 | 2665.5 | 7.7 |
| 26 | 6.9 | 515.0 | 1576.4 | 7.4 |
| 27 | 7.4 | 408.7 | 1217.8 | 7.3 |
| 28 | 4.1 | 277.0 | 879.3 | 7.9 |
| 29 | 5.0 | 248.0 | 426.1 | 7.8 |
| 30 | 6.7 | 735.6 | 1076.6 | 7.8 |
| 31 | 6.2 | 524.8 | 3218.8 | 7.6 |
| 32 | 3.2 | 221.9 | 622.5 | 8.0 |
| 33 | 3.2 | 143.9 | 583.7 | 7.6 |
| 34 | 6.6 | 643.3 | 1361.5 | 7.8 |
| 35 | 2.6 | 152.3 | 507.6 | 7.8 |
| 36 | 7.7 | 380.7 | 1212.3 | 8.2 |
| 37 | 4.0 | 204.3 | 544.2 | 7.4 |
| 38 | 0.0 | 115.2 | 318.4 | 7.3 |
| 39 | 3.0 | 209.7 | 534.3 | 7.5 |
| 40 | 8.1 | 601.8 | 2363.3 | 7.8 |
| 41 | 5.7 | 422.0 | 1114.6 | 7.9 |
| 42 | 6.2 | 490.4 | 1230.5 | 7.8 |
| 43 | 10.2 | 532.3 | 2401.8 | 7.8 |
| 44 | 5.4 | 352.2 | 1059.1 | 7.2 |
| 45 | 7.3 | 413.8 | 1221.1 | 7.8 |
| 46 | 2.0 | 167.9 | 337.4 | 7.6 |
| 47 | 5.6 | 356.9 | 876.6 | 7.5 |
| 48 | 2.4 | 164.5 | 318.4 | 7.3 |
| 49 | 7.1 | 383.5 | 1053.7 | 7.1 |
| 50 | 9.4 | 282.4 | 1257.2 | 7.8 |

**Table 7** Content of Cd, Pb and Zn in soil samples and soil pH from family allotment gardens in **zone** **No. I**, **sampling point No. 2**

| Sample number | Cd  [mg kg^-1^ dry weight] | Pb  [mg kg^-1^ dry weight] | Zn  [mg kg^-1^ dry weight] | pH |
| --- | --- | --- | --- | --- |
| 1 | 3.5 | 179.9 | 280.7 | 7.5 |
| 2 | 8.2 | 289.0 | 1068.2 | 7.4 |
| 3 | 69.9 | 1226.8 | 7443.0 | 7.5 |
| 4 | 16.2 | 760.6 | 1117.0 | 7.5 |
| 5 | 5.9 | 288.4 | 578.2 | 7.9 |
| 6 | 27.6 | 1293.4 | 1966.3 | 7.9 |
| 7 | 24.7 | 765.1 | 2279.3 | 7.9 |
| 8 | 6.2 | 250.8 | 600.0 | 8.1 |
| 9 | 11.8 | 373.2 | 1709.4 | 7.9 |
| 10 | 10.5 | 397.8 | 929.2 | 7.9 |
| 11 | 19.2 | 662.9 | 1515.3 | 7.7 |
| 12 | 39.0 | 1508.8 | 1890.2 | 7.4 |
| 13 | 14.6 | 519.9 | 857.3 | 7.5 |
| 14 | 10.2 | 405.5 | 717.3 | 7.6 |
| 15 | 33.4 | 1223.0 | 1169.3 | 7.4 |
| 16 | 28.5 | 894.5 | 1468.6 | 7.6 |
| 17 | 9.3 | 378.7 | 562.3 | 7.1 |
| 18 | 51.7 | 2777.6 | 2410.5 | 6.9 |
| 19 | 11.2 | 304.7 | 598.8 | 7.1 |
| 20 | 13.8 | 588.1 | 723.0 | 7.2 |
| 21 | 6.7 | 782.2 | 953.1 | 7.8 |
| 22 | 11.2 | 682.8 | 1738.8 | 7.9 |
| 23 | 11.0 | 1077.3 | 1754.4 | 8.4 |
| 24 | 7.0 | 626.9 | 1103.9 | 7.4 |
| 25 | 8.3 | 606.1 | 884.1 | 8.1 |
| 26 | 3.1 | 394.5 | 508.0 | 7.8 |
| 27 | 5.3 | 553.9 | 637.3 | 7.7 |
| 28 | 4.5 | 443.8 | 516.3 | 7.7 |
| 29 | 6.1 | 521.3 | 632.4 | 7.9 |
| 30 | 4.6 | 364.4 | 529.2 | 7.8 |
| 31 | 5.2 | 467.3 | 538.7 | 7.9 |
| 32 | 4.6 | 473.0 | 533.1 | 7.8 |
| 33 | 4.3 | 549.2 | 569.1 | 6.9 |
| 34 | 4.5 | 497.8 | 406.3 | 7.3 |
| 35 | 2.8 | 477.5 | 569.7 | 7.5 |
| 36 | 6.6 | 384.3 | 903.1 | 7.9 |
| 37 | 3.4 | 472.3 | 477.0 | 7.4 |
| 38 | 5.6 | 432.5 | 521.4 | 7.7 |
| 39 | 9.3 | 485.6 | 1139.5 | 7.8 |
| 40 | 13.2 | 593.8 | 1110.7 | 7.5 |

**Table 8** Content of Cd, Pb and Zn in soil samples and soil pH from family allotment gardens in **zone** **No. II**, **sampling point No. 1**

| Sample number | Cd  [mg kg^-1^ dry weight] | Pb  [mg kg^-1^ dry weight] | Zn  [mg kg^-1^ dry weight] | pH |
| --- | --- | --- | --- | --- |
| 1 | 13.5 | 434.2 | 2025.8 | 8.0 |
| 2 | 15.7 | 403.2 | 2094.8 | 7.7 |
| 3 | 10.9 | 462.0 | 1888.0 | 7.9 |
| 4 | 8.1 | 312.8 | 1314.1 | 8.0 |
| 5 | 6.4 | 257.6 | 1017.8 | 8.1 |
| 6 | 18.2 | 1224.4 | 6736.8 | 8.2 |
| 7 | 5.8 | 152.3 | 705.4 | 8.0 |

**Table 9** Content of Cd, Pb and Zn in soil samples and soil pH from family allotment gardens in **zone** **No. III**, **sampling point No. 1**

| Sample number | Cd  [mg kg^-1^ dry weight] | Pb  [mg kg^-1^ dry weight] | Zn  [mg kg^-1^ dry weight] | pH |
| --- | --- | --- | --- | --- |
| 1 | 5.2 | 132.6 | 616.4 | 6.2 |
| 2 | 2.5 | 76.2 | 375.8 | 7.2 |
| 3 | 4.0 | 173.4 | 980.6 | 7.5 |
| 4 | 6.1 | 319.0 | 1062.9 | 7.2 |
| 5 | 14.8 | 478.7 | 2914.3 | 7.5 |
| 6 | 12.6 | 518.7 | 4008.0 | 7.7 |
| 7 | 14.4 | 346.4 | 1927.6 | 8.0 |
| 8 | 7.3 | 258.8 | 770.8 | 8.0 |
| 9 | 4.5 | 158.2 | 554.5 | 8.3 |
| 10 | 4.8 | 172.4 | 590.3 | 8.1 |
| 11 | 3.3 | 145.2 | 592.4 | 7.9 |
| 12 | 4.5 | 220.6 | 917.0 | 8.0 |
| 13 | 5.8 | 212.1 | 933.2 | 7.5 |
| 14 | 9.7 | 287.4 | 1293.5 | 7.3 |
| 15 | 9.2 | 276.6 | 1039.1 | 7.2 |
| 16 | 4.7 | 170.6 | 656.1 | 7.7 |
| 17 | 15.5 | 482.8 | 2367.3 | 7.6 |
| 18 | 9.5 | 187.4 | 885.3 | 8.4 |
| 19 | 15.5 | 453.1 | 1786.8 | 8.4 |
| 20 | 12.7 | 659.0 | 1618.4 | 7.8 |
| 21 | 18.3 | 501.6 | 1826.7 | 7.5 |
| 22 | 11.3 | 289.0 | 1087.6 | 7.5 |
| 23 | 12.8 | 16.8 | 392.6 | 7.4 |
| 24 | 10.4 | 364.0 | 1222.6 | 7.4 |
| 25 | 11.8 | 353.7 | 1297.2 | 7.6 |
| 26 | 6.1 | 198.8 | 911.1 | 7.7 |
| 27 | 8.6 | 239.0 | 1360.2 | 7.4 |

**Table 10** Content of Cd, Pb and Zn in soil samples and soil pH from family allotment gardens in **zone** **No. III**, **sampling point No. 2**

| Sample number | Cd  [mg kg^-1^ dry weight] | Pb  [mg kg^-1^ dry weight] | Zn  [mg kg^-1^ dry weight] | pH |
| --- | --- | --- | --- | --- |
| 1 | 15.7 | 1903.3 | 497.5 | 17.6 |
| 2 | 17.7 | 2123.8 | 512.4 | 11.2 |
| 3 | 15.0 | 1782.8 | 411.5 | 19.3 |
| 4 | 13.4 | 1800.6 | 416.9 | 12.1 |
| 5 | 14.9 | 2278.3 | 499.4 | 12.1 |
| 6 | 16.5 | 2175.7 | 460.3 | 12.6 |
| 7 | 14.3 | 1897.5 | 536.9 | 12.2 |
| 8 | 14.3 | 1920.7 | 480.1 | 13.4 |
| 9 | 15.5 | 1794.9 | 425.1 | 16.9 |
| 10 | 10.6 | 1622.3 | 390.5 | 10.7 |
| 11 | 16.2 | 2195.3 | 569.6 | 13.7 |
| 12 | 14.1 | 2463.3 | 551.5 | 15.5 |
| 13 | 17.9 | 2275.6 | 550.0 | 15.1 |
| 14 | 14.8 | 2000.0 | 502.0 | 23.7 |
| 15 | 13.7 | 1920.4 | 480.4 | 12.7 |
| 16 | 11.3 | 1475.7 | 354.1 | 11.0 |
| 17 | 12.6 | 1507.4 | 375.1 | 11.5 |
| 18 | 16.6 | 1947.5 | 510.9 | 12.1 |
| 19 | 11.7 | 1684.4 | 414.0 | 10.3 |
| 20 | 12.7 | 2219.8 | 418.2 | 20.2 |
| 21 | 15.1 | 1770.0 | 419.8 | 10.1 |
| 22 | 16.7 | 2668.6 | 479.3 | 20.0 |
| 23 | 13.8 | 1524.7 | 394.6 | 10.5 |
| 24 | 14.4 | 1926.4 | 761.6 | 12.0 |
| 25 | 13.0 | 1779.3 | 415.2 | 10.1 |
| 26 | 9.2 | 1484.4 | 350.2 | 6.7 |
| 27 | 16.8 | 1715.6 | 508.5 | 23.7 |
| 28 | 16.6 | 1658.2 | 407.6 | 11.2 |
| 29 | 14.7 | 1732.9 | 395.7 | 9.9 |
| 30 | 14.0 | 1665.3 | 401.4 | 10.2 |
| 31 | 14.8 | 2285.7 | 470.2 | 12.7 |
| 32 | 13.1 | 1781.7 | 401.4 | 9.7 |
| 33 | 16.0 | 1695.9 | 503.3 | 13.6 |
| 34 | 11.4 | 1302.2 | 336.5 | 7.6 |
| 35 | 9.3 | 1546.2 | 325.4 | 9.0 |
| 36 | 17.5 | 2063.5 | 562.7 | 16.0 |
| 37 | 16.0 | 2019.8 | 542.2 | 14.0 |
| 38 | 13.9 | 1773.3 | 425.2 | 12.7 |
| 39 | 13.4 | 2003.9 | 507.8 | 6.9 |
| 40 | 14.9 | 2121.3 | 503.2 | 14.5 |
| 41 | 8.8 | 1426.2 | 336.2 | 12.1 |
| 42 | 14.7 | 2025.6 | 512.8 | 21.2 |
| 43 | 33.1 | 1687.0 | 412.8 | 16.8 |
| 44 | 15.7 | 2045.1 | 546.1 | 11.4 |
| 45 | 17.5 | 2270.4 | 580.4 | 15.9 |
| 46 | 16.3 | 2823.9 | 772.2 | 0.2 |
| 47 | 15.1 | 1943.2 | 473.6 | 14.2 |
| 48 | 14.3 | 1588.4 | 405.7 | 12.5 |
| 49 | 14.9 | 1973.5 | 447.3 | 12.1 |

**Table 11** Content of Cd, Pb and Zn in soil samples and soil pH from family allotment gardens in **zone** **No. IV**, **sampling point No. 1**

| Sample number | Cd  [mg kg^-1^ dry weight] | Pb  [mg kg^-1^ dry weight] | pH |
| --- | --- | --- | --- |
| 1 | 1,4 | 71,2 | 7,1 |
| 2 | 1,4 | 70,6 | 7,8 |
| 3 | 1,7 | 87,4 | 7,5 |
| 4 | 1,8 | 79,2 | 7,6 |
| 5 | 3,3 | 122,6 | 7,3 |
| 6 | 2,2 | 116,2 | 7,5 |
| 7 | 1,2 | 62,7 | 7,6 |
| 8 | 1,8 | 77,7 | 7,6 |
| 9 | 0,0 | 50,2 | 7,5 |
| 10 | 2,3 | 93,5 | 7,9 |
| 11 | 1,3 | 99,3 | 7,3 |
| 12 | 3,6 | 139,3 | 7,6 |
| 13 | 2,7 | 122,8 | 7,9 |
| 14 | 3,0 | 107,9 | 7,9 |
| 15 | 3,0 | 105,1 | 8,1 |
| 16 | 5,3 | 212,0 | 7,8 |
| 17 | 4,8 | 170,2 | 8,1 |
| 18 | 4,8 | 193,7 | 7,7 |
| 19 | 4,4 | 129,1 | 8,0 |
| 20 | 1,2 | 55,8 | 7,4 |
| 21 | 0,0 | 42,5 | 6,6 |
| 22 | 1,6 | 59,9 | 7,6 |
| 23 | 1,6 | 103,4 | 8,0 |
| 24 | 2,2 | 118,8 | 7,1 |
| 25 | 2,7 | 110,7 | 7,9 |
| 26 | 2,4 | 116,2 | 7,0 |
| 27 | 1,4 | 62,3 | 7,8 |
| 28 | 2,2 | 87,5 | 7,7 |
| 29 | 1,7 | 83,2 | 7,7 |
| 30 | 1,6 | 90,7 | 8,2 |
| 31 | 4,6 | 99,0 | 7,6 |
| 32 | 1,8 | 93,5 | 7,9 |
| 33 | 2,6 | 96,2 | 8,0 |
| 34 | 3,3 | 98,2 | 7,9 |
| 35 | 4,2 | 166,7 | 8,0 |
| 36 | 2,5 | 87,4 | 8,5 |
| 37 | 2,5 | 152,6 | 7,6 |
| 38 | 1,4 | 79,9 | 7,0 |
| 39 | 1,4 | 67,2 | 8,1 |
| 40 | 1,7 | 82,6 | 7,9 |
| 41 | 0,0 | 80,6 | 8,1 |
| 42 | 1,4 | 104,3 | 8,2 |
| 43 | 1,4 | 128,2 | 7,1 |
| 44 | 1,4 | 79,4 | 7,4 |
| 45 | 1,5 | 84,4 | 7,8 |
| 46 | 2,0 | 79,7 | 8,0 |
| 47 | 1,4 | 100,0 | 7,6 |
| 48 | 2,5 | 90,4 | 8,0 |

**Table 12** Content of Cd, Pb and Zn in vegetable samples from family allotment gardens

| Vegetable | Sample number | Cd  [mg kg^-1^ fresh weight] | Pb  [mg kg^-1^ fresh weight] | Zn  [mg kg^-1^ fresh weight] |
| --- | --- | --- | --- | --- |
| carrot | 1 | 0.093 | <0.007 | 13.320 |
|  | 2 | 0.330 | 0.034 | 3.420 |
|  | 3 | 0.550 | 1.090 | 21.770 |
|  | 4 | 0.340 | 0.500 | 11.240 |
|  | 5 | 0.370 | 0.860 | 17.800 |
|  | 6 | 0.670 | 1.350 | 28.340 |
|  | 7 | 0.750 | 2.060 | 6.020 |
|  | 8 | 1.120 | 2.120 | 12.170 |
|  | 9 | 0.040 | <0.007 | no data |
|  | 10 | 0.220 | <0.007 | no data |
|  | 11 | 0.080 | <0.007 | no data |
|  | 12 | 0.530 | 0.530 | no data |
|  | 13 | 2.000 | 0.910 | no data |
|  | 14 | 4.820 | 1.480 | no data |
| potato | 1 | 0.230 | 0.300 | 15.110 |
|  | 2 | 0.340 | 0.050 | 9.830 |
|  | 3 | 0.280 | 0.490 | 9.030 |
|  | 4 | 1.700 | 1.650 | 161.560 |
|  | 5 | 0.040 | <0.007 | 0.080 |
|  | 6 | 0.040 | 0.050 | 0.080 |
|  | 7 | 0.010 | 0.050 | 0.030 |
|  | 8 | 0.010 | 0.050 | 0.030 |
|  | 9 | 0.020 | 0.050 | 0.050 |
|  | 10 | 0.030 | 0.050 | 0.060 |
|  | 11 | 0.040 | 0.050 | 0.070 |
|  | 12 | 0.020 | 0.050 | 0.050 |
|  | 13 | 0.030 | 0.050 | 0.050 |
|  | 14 | 0.020 | 0.050 | 0.050 |
|  | 15 | 0.070 | 0.050 | 0.140 |
|  | 16 | 0.030 | 0.050 | 0.070 |
| beetroot | 1 | 0.410 | 0.850 | 72.180 |
|  | 2 | 0.340 | 0.550 | 44.860 |
|  | 3 | 0.510 | 0.620 | 59.260 |
|  | 4 | 0.140 | 0.400 | no data |
|  | 5 | 0.200 | 0.030 | no data |
|  | 6 | 0.590 | 0.540 | no data |
|  | 7 | 0.070 | <0.007 | no data |
|  | 8 | 0.120 | <0.007 | no data |
| parsley | 1 | 0.081 | 0.137 | 11.860 |
|  | 2 | 0.220 | 0.630 | no data |
|  | 3 | 0.250 | 0.980 | no data |
|  | 4 | 0.360 | <0.007 | no data |
|  | 5 | 0.180 | 1.400 | 24.260 |
|  | 6 | 0.370 | 0.700 | 57.710 |
|  | 7 | 0.370 | 2.070 | 44.240 |
|  | 8 | 0.550 | 3.020 | 19.310 |
|  | 9 | 0.690 | 5.540 | 34.520 |
|  | 10 | 0.020 | <0.007 | no data |
|  | 11 | 0.020 | <0.007 | no data |
|  | 12 | 0.010 | <0.007 | no data |
|  | 13 | 0.130 | <0.007 | no data |
| celery | 1 | 0.459 | 0.271 | 26.150 |
|  | 2 | 0.310 | <0.007 | no data |
|  | 3 | 1.110 | 0.800 | no data |
|  | 4 | 2.820 | 0.520 | no data |
|  | 5 | 4.540 | 0.340 | no data |
|  | 6 | 0.100 | <0.007 | no data |
|  | 7 | 0.250 | <0.007 | no data |
|  | 8 | 0.170 | <0.007 | no data |
|  | 9 | 0.190 | <0.007 | no data |
|  | 10 | 0.280 | <0.007 | no data |
|  | 11 | 0.170 | <0.007 | no data |
|  | 12 | 0.950 | 1.770 | 46.400 |
|  | 13 | 1.650 | 1.650 | 89.480 |
